# Identifying Priority Areas for Increasing the Supply of Medication-Assisted Treatments for Opioid Use Disorder: A Geospatial Approach

**DOI:** 10.36469/9787

**Published:** 2018-04-16

**Authors:** Michael Topmiller, Peter J. Mallow, Aaron T. Vissman, Jene Grandmont

**Affiliations:** 1 HealthLandscape LLC, American Academy of Family Physicians, Cincinnati, OH, USA; 2 Xavier University, Cincinnati, OH, USA; 3 Center for Health and Human Services Research, Talbert House, Cincinnati, OH, USA; 4 HealthLandscape LLC, American Academy of Family Physicians, Cincinnati, OH, USA

**Keywords:** Opioid Use Disorder, Illicit Drug Dependence, Geospatial Analysis, Medication-Assisted Treatment, United States, Substance Abuse

## Abstract

**Background:**

The opioid epidemic has disproportionately affected several areas across the United States (US), with research indicating that these areas may be underserved and lack access to sufficient medication-assisted treatment (MAT) options. The objective of this study was to introduce a geospatial analytical framework for identifying sub-state priority areas to target federal allocation of MAT training and resources.

**Methods:**

We used a geospatial analytical framework, which integrated multiple substance use measures and layers of geographic information. Measures included estimates of illicit drug dependence and unmet treatment need from the National Survey on Drug Use and Health (NSDUH), opioid-related admissions from the Treatment Episode Data Set: Admissions (TEDs-A), and Drug Enforcement Agency (DEA) waiver practitioner data from the Substance Abuse and Mental Health Services Administration (SAMHSA). Analyses included standard deviation outlier mapping, local indicators of spatial autocorrelation (LISA), and map overlays.

**Results:**

We identified twenty-nine opioid dependence priority areas, eleven unmet treatment need priority areas, and seven low MAT capacity priority areas, located across the US, including southeastern Ohio, western Indiana, the District of Columbia, New England, and northern and southern California.

**Conclusions:**

This study identified several areas across the US that have unmet need for MAT. Targeting these areas will allow for the most effective deployment of cost-effective MAT resources to aid the greatest number of patients with opioid use disorders.

## Introduction

Almost 3 million people in the US are affected by substance use disorders related to opioid derived pain relievers (legal or illegal) or heroin, and most are not receiving any kind of medication-assisted treatment (MAT).^1^ Treating opioid use disorders (OUD) with MAT which includes counseling has been shown to reduce mortality, infectious diseases, and other harms of opioid addiction.^2^ Further, MAT has been shown to be one of the most cost-effective treatments for OUD.^3^,^4^,^5^

The benefits of MAT led the United States (US) Department of Health and Human Services (HHS) to direct funding and resources to increase MAT capacity.^6^ Physicians have been able to obtain the Drug Enforcement Agency (DEA) waiver allowing them to treat a specified number of patients (30, 100, 275) with OUDs using buprenorphine since 2002, and more recently, non-physician practitioners (e.g., nurse practitioners, physician assistants) have been able to receive the DEA waiver.^6^–^11^ While a growing number of practitioners are receiving the DEA waiver nationally, the overall percentage is still small. In many areas there are no practitioners with a DEA waiver to treat OUD patients.^12^,^13^

The potential to treat OUD is dependent on increasing MAT capacity by approving more DEA waiver practitioners, and providing additional education and training to those that already have a waiver. Further, place matters in disparities research, and efforts to increase MAT capacity should benefit the highest need areas.^14^ The purpose of this study was to introduce a geospatial analysis framework to identify priority areas for improving MAT capacity for OUD using combinations of substance use data and layers of geographic information.

## Methods

### Data

Data from the National Survey on Drug Use and Health (NSDUH), Treatment Episode Data Set: Admissions (TEDs-A), and the Drug Enforcement Administration (DEA) waiver practitioner data were used in this study. The Substance Abuse and Mental Health Services Administration (SAMHSA) administered the collection and publication of these data sets.^15^

The NSDUH is a national health survey designed to provide national estimates on the use of illicit drugs, abuse and misuse of prescription drugs, alcohol, and tobacco, as well as mental health concerns in the US population.^16^ The estimates for illicit drug dependence and unmet treatment need at the sub-state (n = 362) were the primary units of analysis for this study. The Treatment Episode Data Set: Admissions (TEDs-A) is a national data system of all publicly and most privately funded admissions for treatment of substance use, including misuse and abuse of prescription opioids, disorders in the US.^17^ We aggregated the total number of admissions and the primary substance use problem by core-based statistical area (CBSA). Next, we identified all CBSAs where opioid-related drugs (Heroin or Other Opiates and Synthetics, legal or illegal) had the highest percentage as the primary substance abuse problem as cause of admission. We normalized these estimates based on the population. CBSAs with less than 10 admissions were suppressed and therefore removed from the analysis.

### Analysis

Prior to identifying priority areas, treatment capacity of the sub-state regions need to be estimated. We used address-level Drug Enforcement Administration (DEA) waiver practitioner data from SAMHSA.^18^ DEA waiver practitioner addresses were geocoded to the NSDUH sub-state areas to get a count of DEA waiver practitioners for each area. Further, the publicly available files did provide the number of patients each practitioner was certified to treat (30, 100, or 275). Thus, we treated each practitioner equally. MAT capacity was defined as the number of practitioners with a DEA waiver per 100 000 population. When exploring estimates for treatment capacity, we initially included opioid treatment programs (OTPs). However, we decided to focus only on the DEA waivered practitioners as opioid treatment programs must be certified by the DEA and we would have double counted practitioners at OTPs.

To identify the high-need priority areas we used standard deviation (outlier) maps and the Local Moran’s I. Standard deviation maps allow for the identification of areas two standard deviations above the mean for illicit drug dependence, which are outliers in the data.^19^ Local Moran’s I, a local indicator of spatial autocorrelation (LISA) that identifies local clusters which are significantly different from random patterns, was used to identify illicit drug dependence hot spots.^20^

To conduct the Local Moran’s I analysis, we first created a weight matrix that defined the spatial relationship between NSDUH areas. We used queen contiguity, meaning that neighbors were defined if they shared a boundary or vertex with another area. A Local Moran’s I score was produced for illicit drug dependence for each area based on how similar an area’s rates were with its neighbors. Local Moran’s values closer to 1 mean that an area has similar rates as its neighbors, and values close to −1 mean that an area has dissimilar rates from its neighbors. For example, an area with a high rate of illicit drug dependence surrounded by areas with high rates of illicit drug dependence is considered a hot spot. Statistical significance is determined by comparing its observed Moran’s I score with a series of randomly generated scores, which we would expect to be close to zero (i.e., we wouldn’t expect randomly generated rates for areas to produce spatial patterns).

We identified a set of high-need priority areas based on illicit drug dependence rates and opioid-related treatment admission rates. First, we identified outlier areas based on rates of past year illicit drug dependence that were one standard deviation above the mean. Second, we identified clusters of high rates of illicit drug dependence (hot spots). We defined Illicit Drug Dependence Priority Areas (IDD-PAs) as areas that were illicit drug dependence outliers and were part of or adjacent to an illicit drug dependence hot spot. In order to focus on opioid-related illicit drug dependence, we used map overlays to identify IDD-PAs that were within or adjacent to CBSAs with opioid-related drugs as their primary substance use problem. We defined these areas as Opioid Dependence Priority Areas (OD-PAs). We explored treatment capacity by identifying OD-PAs with the highest unmet treatment need and lowest MAT capacity. OD-PAs that were two standard deviations above the mean for unmet treatment need for illicit drug dependence were considered Unmet Treatment Need Priority Areas (UTN-PAs), while Low MAT Capacity Priority Areas (LMATC-PAs) were defined as OD-PAs in the bottom 50th percentile for DEA waiver practitioners per 100 000 population (See Table 1 for criteria defining priority areas). All analyses were conducted with GeoDa, an open-source software package for geospatial analysis and visualization.^21^

## Results

**Figure 1:**
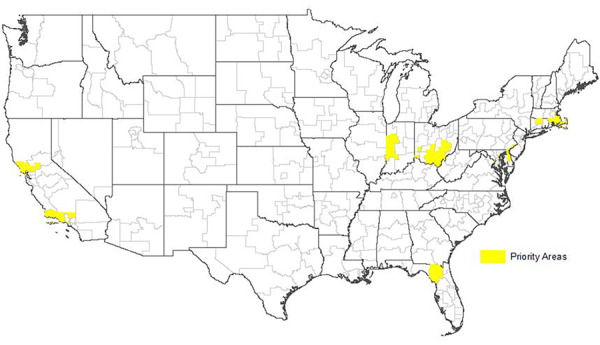
OD-PAs The 29 OD-PAs identified in our analysis are shown in yellow. OD-PA: opioid dependence priority area

Our analyses identified 32 IDD-PAs that have rates of illicit drug dependence at least one standard deviation above the mean and are located within or adjacent to illicit drug dependence hot spots. By overlaying CBSAs where opioid-related drugs were the primary reason for treatment admission, we found that 29 areas were identified as OD-PAs. Figure 1 displays the OD-PAs, which can be found in eleven states across the US, with more than half located in urban areas in the northeastern US, including seven of the District of Columbia’s eight wards.

**Figure 2:**
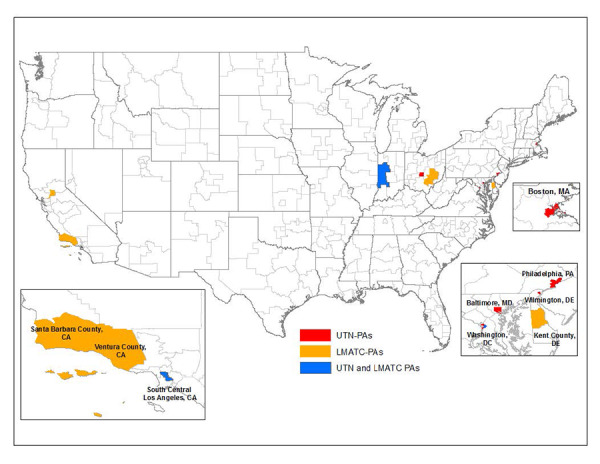
UTN-PAs & LMATC-PAs The UTN-PAs are shown in red. The LMATC-PAs are shown in orange, and the sub-state areas that were identified as both UTN-PA and LMATC-PA are shown in blue. LMATC-PA: low medication-assisted treatment capacity priority area; OD-PA: opioid dependence priority area; UTN-PA: unmet treatment need priority area

While most OD-PAs have high-unmet treatment need rates relative to the national average, we identified eleven UTN-PAs, which have unmet treatment need rates more than two standard deviations above the mean. With the exception of West Indiana, all of the UTN-PAs are located in urban areas, primarily in the Northeast, but also in Los Angeles, CA and Columbus, OH (see Figure 2).

There were seven OD-PAs in the bottom 50th percentile for MAT capacity among all areas, as displayed in Figure 2. These Low MAT Capacity Priority Areas (LMATC-PAs) are diverse and located in rural (West Indiana and Southeast Ohio), large urban (Los Angeles, Washington DC, and Sacramento), and small urban areas (Dover County, DE, and Santa Barbara/Ventura, CA).

**Figure 3:**
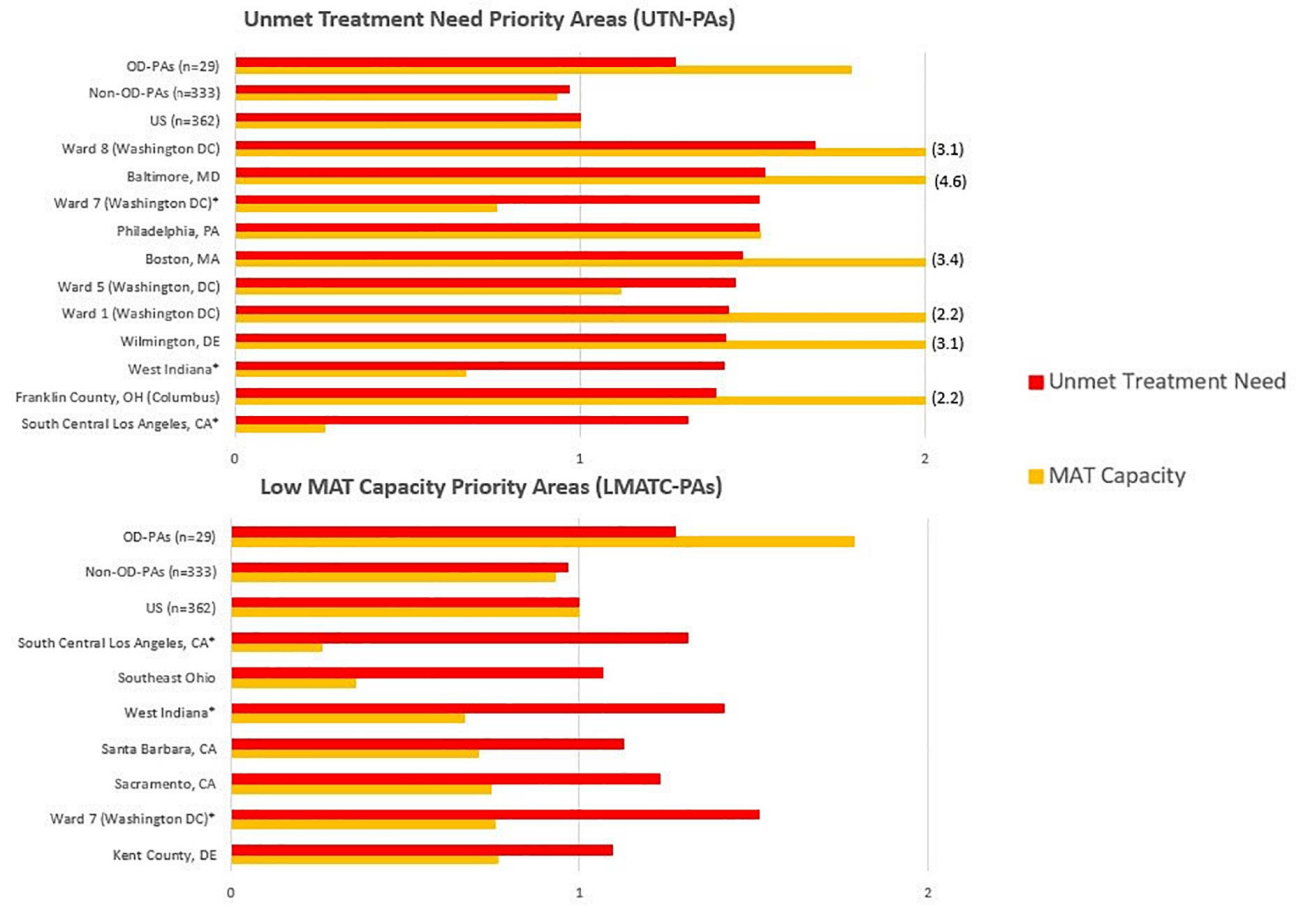
Characteristics of UTN-PAs, LMATC-PAs, and OD-PAs Relative to National Averages The figure shows the unmet treatment need and MAT capacity relative to the national average. A score of 1 equals the national average. A score higher than 1 indicates the need is higher in the specific region relative to the national average. LMATC-PA: low medication-assisted treatment capacity priority area; MAT: medication-assisted treatment; OD-PA: opioid dependence priority area; UTN-PA: unmet treatment need priority area

In general, we found that UTN-PAs have higher MAT capacity than the average area, but with varying rates of capacity (see Figure 3). For example, Baltimore has one of the highest unmet treatment need rates and the highest MAT capacity (almost 5 times the national rate), while Philadelphia has the third highest unmet treatment need and much lower MAT capacity, though still in the top 50th percentile among all areas. In addition, West Indiana, South Central Los Angeles, and Ward 7 (Washington DC) are both UTN-PAs and LMATC-PAs.

While OD-PAs have higher rates of MAT capacity, they may also be considered uniquely high burden areas and, in general, have high rates of unmet treatment need for illicit drug dependence compared with non-OD-PAs and the United States overall (Figure 3).

To our knowledge, this study is the first to identify priority areas for targeting MAT capacity with respect to the opioid epidemic at the sub-state level. Our geospatial analytical framework was established to identify sub-state areas that may have the greatest number of people dealing with OUD and least resources available to treat this demand. Using our framework to target scarce resources in these 29 sub-state areas may lead to the most efficient allocation of scarce resources. However, by design this framework does not consider equity. If the US Department of HHS targeted all available resources to these 29 areas it is very possible some high poverty areas may not have any treatment options available. The question of helping the greatest number of people or providing some minimum level of treatment access to all is beyond the scope of this study.

Two studies have previously explored the allocation of MAT treatment capacity.^27^,^28^ Jones *et al*. identified gaps in MAT capacity and need at the state and national level but did not explore variation within states or cross-border travel that occurs in many sub-state areas.^27^ Abraham *et al*. explored county-level geographic variation in MAT treatment from opioid treatment programs (OTPs), finding gaps in treatment in areas with high rates of OUDs in the southeastern United States. However, this study only explored opioid treatment programs accepting Medicaid and did not include physicians treating OUD.^28^

## Discussion

We found 29 OD-PAs that may warrant increased access to cost-effective MAT options to mitigate the opioid epidemic. These OD-PAs are located predominantly in states ranked in the top 10 for drug positioning mortality rates, such as Ohio (ranked 2nd), New Hampshire (ranked 3rd), Pennsylvania (ranked 5th), Massachusetts (ranked 8th), and Rhode Island (ranked 9th).^22^

When unmet treatment need and MAT treatment capacity were factored into the analysis, we identified several areas with high unmet treatment need and low MAT treatment capacity. Adequate response and efficient use of scarce resources will require targeting high-need areas with increased numbers of practitioners with DEA waivers and additional resources and training for those that already have a waiver. Research has indicated that many physicians do not treat OUD patients due to several barriers, including stigma, inadequate reimbursement, and a lack of training, resources, and institutional support.^11^,^12^,^23^–^26^

Overcoming barriers related to stigma, reimbursement, and institutional support will require focused effort and allocation of resources. However, increasing practitioners’ access to training and resources is relatively straight forward, and tailored strategies target specific geographic areas will provide a more efficient allocation of resources.

## Limitations

This novel approach to identify specific areas to allocate scarce MAT resources for the treatment of OUD is not without limitations. First, we combined several publicly available data sources to identify the OD-PAs. This required us to use the lowest common denominator approach to join the data. As such, granularity of all available data is lost. The public use files are already constrained and we were required to use illicit drug dependence estimates for sub-state areas, which included non-opioid related drug dependence. Though, by overlaying the TEDS-A data we found that OUD was the most likely substance abuse problem. Second, in many regions treatment admission data were suppressed due to counts of OUD <10. We imputed the missing data by using the illicit drug dependence treatment admission data to provide a national picture of priority areas. We ran the analysis without this imputation and our results did not materially change. Third, our data did not include those jailed for crimes or currently institutionalized for drug dependence. If we assume all drug dependent individuals in jail or institutionalized were receiving treatment then our findings hold. However, we do not have the necessary data to make this assumption. Finally, our outlier maps were defined by one standard deviation from the mean. This was a necessarily arbitrary choice. We chose this definition to be more inclusive than if we had used a narrower definition such as two standard deviations from the mean.

The majority of the noted limitations may be mitigated through access to the raw data, which was not currently available. These limitations notwithstanding, this study identifies several areas that may disproportionately benefit from the allocation of cost-effective solutions to address OUD.

## Conclusion

This study illustrated the use of a novel geospatial method to identify opioid dependence priority areas. Focusing on priority areas will maximize the benefit and affordability of providing cost-effective treatments for illicit drug dependence.

## Figures and Tables

**Table 1:** Criteria for Defining PAs

PAs	Criteria	Number of PAs
IDD-PA	Areas that have IDD rates one standard deviation above the mean AND are an IDD hot spot or adjacent to a hot spot	32
OD-PA	IDD-PAs within or adjacent to CBSAs where opioid is primary substance use problem	29
UTN-PAs	OD-PAs with unmet treatment need two standard deviations above the mean	11
LMATC-PAs	OD-PAs with MAT capacity in bottom 50th percentile	7

CBSA: core-based statistical area; IDD: illicit drug dependence; LMATC: low medication-assisted treatment capacity; MAT: medication-assisted treatment; OD: opioid dependence; PA: priority area; UTN: unmet treatment need

## Abbreviations

MAT: Medication-Assisted Treatment
OUD: Opioid Use Disorder
HHS: United States (US) Department of Health and Human Services
DEA: Drug Enforcement Agency
NSDUH: National Survey on Drug Use and Health
TEDs-A: Treatment Episode Data Set: Admissions
SAMHSA: Substance Abuse and Mental Health Services Administration
CBSA: Core-based Statistical Area
LISA: Local Indicator of Spatial Autocorrelation
IDD-PAs: Illicit Drug Dependence Priority Areas
OD-PAs: Opioid Dependence Priority Areas
UTN-PAs: Unmet Treatment Need Priority Areas
LMATC-PAs: Low MAT Capacity Priority Areas
OTPs: Opioid Treatment Programs
